# Potential application of rLc36 protein for diagnosis of canine visceral leishmaniasis

**DOI:** 10.1590/0074-02760170171

**Published:** 2018-03

**Authors:** Camila Tita Nogueira, Mayara Lúcia Del Cistia, Ana Carolina Urbaczek, Márcia MG Jusi, Angela Maria Arenas Velásquez, Rosângela Zacarias Machado, Henrique Ferreira, Flávio Henrique-Silva, Hélio Langoni, Paulo Inácio da Costa, Márcia AS Graminha

**Affiliations:** 1 Universidade Estadual Paulista, Instituto de Química, Campus de Araraquara, Araraquara, SP, Brasil; 2 Universidade Estadual Paulista, Faculdade de Ciências Farmacêuticas, Campus de Araraquara, Araraquara, SP, Brasil; 3 Universidade de São Paulo, Instituto de Química, Campus de São Carlos, São Carlos, SP, Brasil; 4 Universidade Estadual Paulista, Faculdade de Ciências Agrárias e Veterinárias, Campus de Jaboticabal, Jaboticabal, SP, Brasil; 5 Universidade Estadual Paulista, Instituto de Biociências, Campus de Rio Claro, Rio Claro, SP, Brasil; 6 Universidade Federal de São Carlos, Departamento de Genética e Evolução, São Carlos, SP, Brasil; 7 Universidade Estadual Paulista, Faculdade de Medicina Veterinária e de Zootecnia, Campus de Botucatu, Botucatu, SP, Brasil

**Keywords:** canine visceral leishmaniasis, immunodiagnosis, recombinant protein

## Abstract

Visceral leishmaniasis (VL) is fatal if left untreated. Infected dogs are important reservoirs of the disease, and thus specific identification of infected animals is very important. Several diagnostic tests have been developed for canine VL (CVL); however, these tests show varied specificity and sensitivity. The present study describes the recombinant protein rLc36, expressed by *Leishmania infantum*, as potential antigen for more sensitive and specific diagnosis of CVL based on an immunoenzymatic assay. The concentration of 1.0 μg/mL of rLc36 enabled differentiation of positive and negative sera and showed a sensitivity of 85% and specificity of 71% (with 95% confidence), with an accuracy of 76%.

Kala azar, or visceral leishmaniasis (VL), is a zoonosis caused by protozoan parasites of the *Leishmania donovani* complex. *L. donovani* and *L. infantum* are the etiological agents of VL in the Old World and *L. infantum* in the Americas (Ikonomopoulos et al. 2003). In Latin America, VL is transmitted by the bite of infected sand flies *Lutzomyia longipalpis* (Akhoundi et al. 2016), with domestic dogs (*Canis familiaris*) as the main reservoir for leishmaniasis infection. Thus, one of the most important approaches for controlling the incidence of human VL is to identify infected dogs (Lira et al. 2006).

Clinical diagnosis of canine VL (CVL) is difficult to achieve because of the nonspecific symptoms in common with other diseases (Mondal et al. 2010). Parasitological methods are based on biopsy or aspiration specimens, but these methods are invasive and present variable sensitivity (Silva et al. 2014).

Among the different serological tests available, enzyme-linked immunosorbent assay (ELISA), indirect immunofluorescence, direct agglutination test, dot-ELISA, and western blotting are the most widely used (Silva et al. 2014). Several *Leishmania* antigens have been evaluated for serodiagnosis (Badaró et al. 1996, Jensen et al. 1999, Akhoundi et al. 2013), with variable sensitivity and specificity.

Here, we characterised the rLc36 protein encoded by the *L. infantum* gene *LinJ.36.4190* as a potential antigen for developing a more sensitive and specific test for CVL based on ELISA.

*Lc36* (GenBank: *LinJ.36.4190*) was identified through bioinformatic analyses using the EupathDB (eupathdb.org) and TriTrypDB (tritrypdb.org) databases. B-Cell epitope prediction was performed using web server-based software (Saha & Raghava 2006), and the B-cell epitope database Bcipep (Saha et al. 2005; available from: http://www.imtech.res.in/raghava/bcipep). Hydropathicity and the presence of transmembrane domains were also analysed (Kyte & Doolittle 1982, Cserzo et al. 2004).

*L. infantum Lc36* codes for a protein of 733 residues that does not contain transmembrane domains (Supplementary data, Fig. 3). Its product is similar to the proteins of *L. donovani* and *Leishmania mexicana*; however, when compared to *Leishmania braziliensis*, *Lc36* shows sequence conservation only at the DNA level, indicating that *Lc36* is a pseudogene in *L. braziliensis* (Supplementary data, Fig. 1A-B). Lc36 did not present any sequence similarity when searched against other genome databases including those of *Crithidia*, *Leptomonas*, *Endotrypanum*, and *Trypanosoma*.

Quantitative polymerase chain reaction (PCR) assays were performed to assess the gene expression levels in different parasite life-cycle stages. For both quantitative and expression assays, we used *L. infantum* strain MHOM/BR/1972/LD, which was donated by the Instituto Oswaldo Cruz (FIOCRUZ, Rio de Janeiro, Brazil). The parasites were cultivated as promastigotes in LIT (Liver Infusion Tryptose - BD Bioscience, San Jose, CA, USA) medium supplemented with 10% foetal bovine serum at 26°C, pH 7.2.

For quantitative PCR assays, we used mouse peritoneal macrophages collected from adult male Swiss albino mice, infected with *L. infantum* as previously described (Velásquez et al. 2017), except that the assay was performed in 25-cm^2^ cell culture flasks (Nunc™, Roskilde, Denmark). These assays were approved by the Ethics Committee for Animal Experimentation (Protocol CEUA/FCF/CAr no. 40/2015) in agreement with Sociedade Brasileira de Ciência de Animais de Laboratório and Conselho Nacional de Controle de Experimentação Animal.

For RNA extraction and real-time PCR analysis, primers RT_36_Forward 5′-GTTCGTCACCGTTGTCTTC-3′ and RT_36_Reverse 5′-GTCGTG CTTCCTGCTATTC-3′ were designed using the software tools GeneRunner (http://www.generunner.com) and Primer Express version 3.0. Total promastigotes and infected and non-infected macrophage RNA were extracted using TRIzol® Reagent (Life Technologies, Carlsbad, CA, USA) according to the manufacturer's protocol. Intracellular amastigote RNA was extracted at 18, 48, and 72 h post-infection. For cDNA synthesis, we used 1 μg of total RNA, the 3′Race System for Rapid Amplification of cDNA ends kit (Life Technologies), and random hexamer primers. Real-time PCR as-says were carried out in a StepOnePlus™ Real-Time PCR System v2.3 (Applied Biosystems, Foster City, CA, USA) with initial denaturation at 95°C for 20 s followed by 40 denaturation cycles at 95°C for 15 s, annealing at 60°C for 1 min, and extension at 95°C for 15 s. For each reaction, 1x Fast Power SYBR® Green Master Mix (Applied Biosystems), 0.1 μM of each primer, and 10 ng of each cDNA sample was added in 10-μL of PCR mixture. A 10-fold dilution series of plasmid DNA (recombinant plasmid pET28a+_Lc36) was used for absolute standard curve construction to determine the efficiency. Assays were carried out in triplicate. Absolute amounts of PCR products were determined based on the threshold cycle (*C*t), by interpolating each *C*t value from each sample on the corresponding standard curve and by the size of plasmid DNA template (7588 base pairs) using the equation described in the Supplementary data (Equation 1). For graphics design, we used software Origin® and Excel® software. These assays revealed that the gene is likely expressed in amastigote cells, the form responsible for the disease's clinical manifestations. The absolute standard curve parameters showed an efficiency of 99.23% and linear regression coefficient (R^2^) of 0.98 (Supplementary data, Table II, Figs 5, 6). Fig. 1 shows that the cDNA from intracellular amastigotes of 18 h and 48 h post-infection macrophages had C_T_ values of 36.0 ± 0.8 and 32.2 ± 0.6, respectively, while C_T_ values higher than 40 were observed for cDNA from promastigotes and intracellular amastigotes at 72 h post-infection. These data were converted to cDNA copy numbers (Supplementary data, Table II, Figs 5-7) and Fig. 1 illustrates that the cDNA copy number was higher in intracellular amastigotes at 48 h post-infection (20 copies per reaction) compared to in parasites at 18 h post-infection (1 copy per reaction). Thus, the results suggest that this gene has a stage-specific expression pattern and indicate that *L. infantum* Lc36 is amastigote-stage specific and is expressed at 48 h post-infection.

**Fig. 1:**
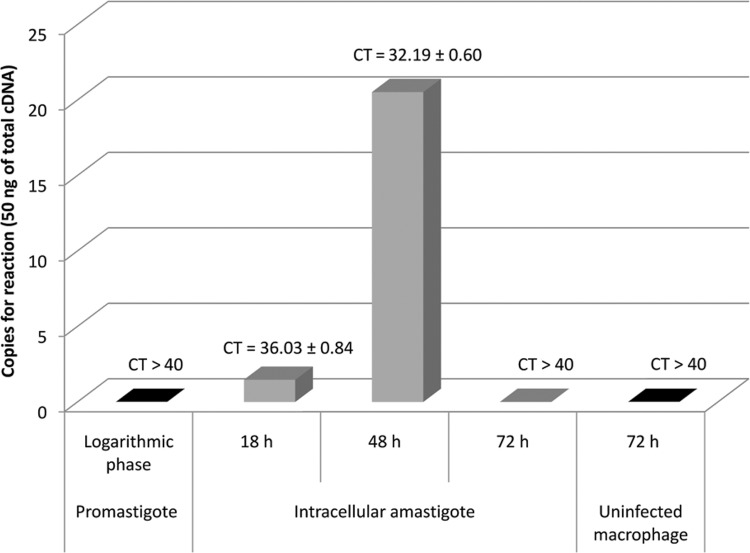
quantitative analysis of Lc36 gene expression in different life-cycle stage forms of *Leishmania infantum*.

Source: authors.

*Leishmania* has an intracellular life stage and is likely to encounter reactive oxygen species produced by the macrophage, which can induce DNA damage. Thus, stage-specific amastigote genes may be involved in parasite survival in macrophages (Genois et al. 2014). Interestingly, *Lc36* codes for a protein of unknown function containing a nucleotidyl transferase domain which is possibly involved in nucleic acid metabolism, including DNA repair, which may play an important role in protecting *Leishmania* amastigote genome integrity during its exposure to reactive oxygen species produced by macrophages (Vaňáčová et al. 2005). For application and diagnosis, some amastigote stage-specific proteins present antigenic properties recognised by the host, such as the A2 protein, which has been evaluated for VL serodiagnosis and vaccine application (Coelho et al. 2012, Martins et al. 2015). As these data indicated the importance of rLc36 for the development of VL diagnosis assays, we tested the performance in ELISA. We mapped linear B cell epitopes within the Lc36 coding region. The protein region from residues 1 to 255 was selected and evaluated for serodiagnosis of CVL (Supplementary data, Table I, Fig. 2). The chosen fragment containing 765 base pairs was amplified and cloned into pET28a+ for heterologous expression in *Escherichia coli.* For expression and purification of rLc36, a recombinant plasmid named as pET28a_Lc36 was constructed (cloning strategy, in Supplementary data). Protein expression was carried out in a 250-mL bacterial culture in LB medium containing 25 μg/mL kanamycin, at 250 rpm and 37°C. The expression of rLc36 was induced by 0.5 mM IPTG and growth was continued at 30°C for 4 h. Next, a bacterial extract was prepared, and the recombinant protein was purified by affinity chromatography using Poly-Prep Chromatography columns (Bio-Rad, Hercules, CA, USA) with the resin Chelating Sepharose TM Fast Flow (GE Healthcare, Little Chalfont, UK). The recombinant protein was eluted with an imidazole gradient ranging from 20 to 200 mM. The His_6_-tagged fusion protein (rLc36), presenting the expected molecular weight, was successfully purified (Supplementary data, Fig. 4).

rLc36 was subjected to 12% SDS-PAGE and transferred to nitrocellulose membranes. Membranes were blocked with 5% non-fat dry milk in 1x PBS, pH 7.3, and incubated overnight at 4°C. The membrane was washed with 0.05% Tween 20 in PBS and incubated for 2 h in the presence of monoclonal anti-polyhistidine 1:1500 (Sigma, St. Louis, MO, USA). After washing, the membrane was treated for 1 h with peroxidase-conjugated anti-mouse secondary antibody (1:40,000), and subsequently developed successively with luminol [50 mL Tris-HCl 1 M pH 8.5, in H_2_O, 5 mL of solution A (0.22 g luminol in 5 mL DMSO), 2.2 mL of solution B (0.033 g of pcoumaric acid, 2.2 mL of DMSO)], and 0.3% H_2_O_2_. The membranes were placed in contact with a photographic film for 5 min and bands were visualised by the addition of Step™ NBT/BCIP (Pierce, Rockford, IL, USA). Western blotting analyses revealed the presence of the purified His_6_-tagged protein in the 200 mM imidazole-eluate soluble fraction and in the inclusion bodies (Fig. 2A).

**Fig. 2:**
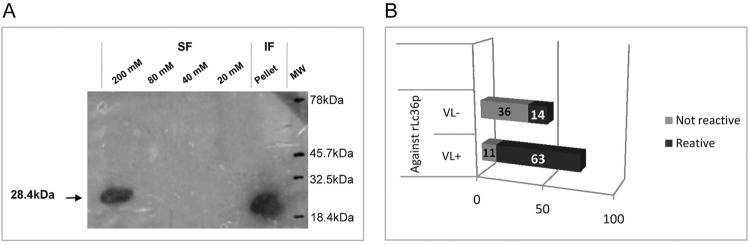
purification and reactivity of protein rLc36. (A) Western blot analysis of purified His6-tagged rLc36. rLc36 was expressed in *Escherichia coli* BL21(DE3) after isopropyl β-d-thiogalactopyranoside induction for 4 h and purified by nickel-affinity chromatography using a gradient of imidazole (20, 40, 80, and 200 mM). The purified recombinant protein was assessed by western blotting using anti-histidine mouse antibody. Non-soluble fraction (IF) obtained after cell lysis and before column purification step. Protein molecular weight determined by Kaleidoscope. (B) Reactivity of antigens rLc36. The cut-off used was 0.191, for a confidence of 95%, using 20 negative sera for visceral leishmaniasis.

Source: authors.

For ELISA, 96-well plates were coated with the recombinant protein rLc36 in 100 μL of coating buffer for 18 h at 4°C. For this assay, 1 μg/mL of rLc36 was used, based on a titration curve developed to determine the optimal recombinant protein concentration. VL-positive sera used in this study were obtained from the area of Campo Grande, Minas Gerais, Brazil, in which CVL is endemic, whereas negative sera were obtained from Jaboticabal, São Paulo, Brazil, a non-endemic area. The sera were selected for CVL based on *Leishmania chagasi*-specific total IgG and *L. chagasi*-specific IgG subclass ELISAs with a confidence index of 95%. Both sera groups were confirmed by direct parasitological methods and indirect immunofluorescence. In the ELISA for rLc36, the plates were blocked with 2% non-fat dry milk solution for 1 h at 37°C. After three washes with PBS Tween 80 at 0.05%, the plates were incubated with 100 μL of canine sera for 1 h at 37°C. Sera samples were diluted 1:200 in PBS with 2% normal rabbit serum. Plates were then washed three times with PBS Tween 80 and incubated with 1:4000 of alkaline phosphatase conjugated to anti-dog IgG (Sigma) for 1 h at 37°C. Plates were washed and 100 μL diethanolamine solution (pH 9.8) containing a substrate for phosphatase (4-nitrophenyl phosphate disodium salt hexahydrate, Sigma) was added to each well. The optical density was measured at 405 nm in an ELISA microplate spectrophotometer (Robonik® - Readwell Touch Automatic ELISA Plate Analyser). Sensitivity and specificity values were determined by discriminating the absorbance data of sera true-positives and true-negatives, as well as false-positives and false-negatives, using cut-off method analysis described by Frey et al. (1998). The accuracy predictive value was also determined (Kawamura 2002). For a complete description, see Supplementary data (Equations 2, 3, 4). The His_6_-tagged protein, confirmed as rLc36, was analysed for antigenic potential against canine sera; 74 sera were previously demonstrated to be positive for CVL while another 50 were negative for this disease (Fig. 2B). Sensitivity and specificity of the rLc36 against the CVL sera were 85% and 72%, respectively, with a cut-off with 95% confidence. Another group of 39 sera samples was separated in symptomatic (n = 33) and asymptomatic (n = 6) subgroups and tested with a cut-off with 99% confidence (Fig. 3). All symptomatic sera were confirmed to be positive for CVL, while for asymptomatic sera, the ELISA based on rLC36 detected four of six asymptomatic samples (Supplementary data, Table V).

**Fig. 3:**
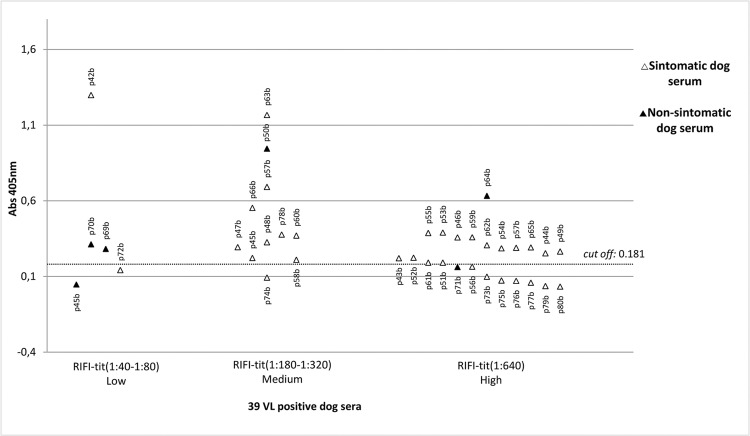
reactivity of 39 positive sera for canine visceral leishmaniasis (CVL) versus rLc36. The dotted line refers to a cut-off of 0.181, calculated based on a confidence of 99%, using 20 negative sera for VL.

Source: authors.

Several recombinant proteins have shown variable effectiveness for leishmaniasis serodiagnosis (Badaró et al. 1996, Bhatia et al. 1999, Jensen et al. 1999, Raj et al. 1999) and others present high amino acid sequence homologies to human and/or canine counterparts, interfering with the applicability of the tests (Maalej et al. 2003, Coelho et al. 2012, Celeste et al. 2014). Thus, based on BLAST analyses revealing a lack of homology to other parasitic sequences, rLc36 may be useful for developing a new and specific diagnostic test for CVL.

In conclusion, the rLc36 protein, which is likely expressed in amastigote forms of *L. infantum*, was capable of differentiating positive from negative CVL sera and showed a sensitivity and specificity of 85% and 71%, respectively, with a confidence of 95% and accuracy of 76%, in a randomly chosen population. The protein was also able to identify asymptomatic animals. These results indicate that rLc36 is promising for CVL serodiagnosis.
