# Supplementary material for: Potential application of rLc36 protein for diagnosis of canine visceral leishmaniasis
Source: Mem Inst Oswaldo Cruz. 2018 Mar;113(3):197–201. doi: 10.1590/0074-02760170171 (PMC5804312; doi:10.1590/0074-02760170171)
Supplement: Supplementary file 1 [file 0074-0276-mioc-113-03-0197-Suppl02.pdf]

SEQUENCES ANALYSIS

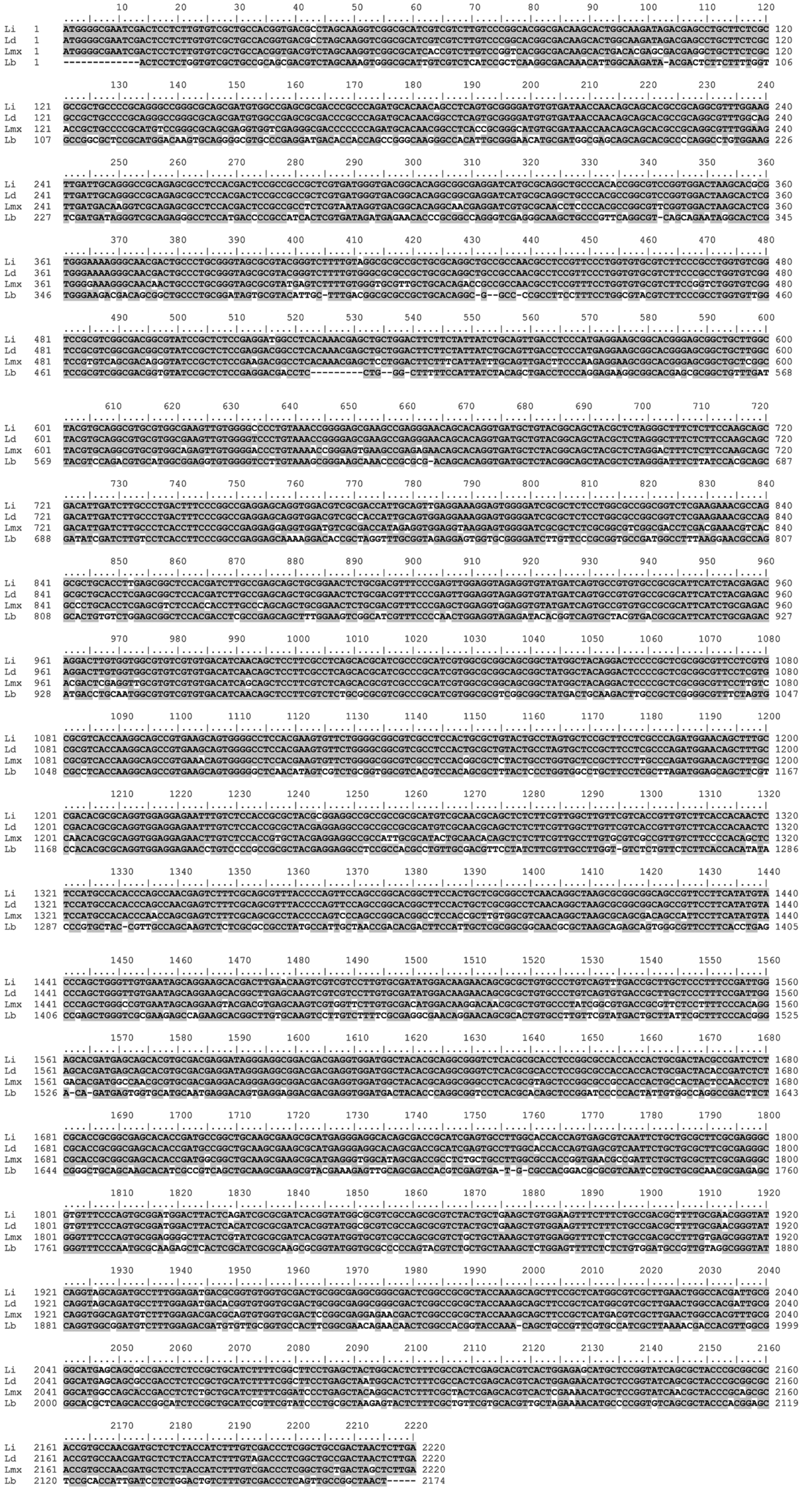

Fig. 1A: multiple nucleotide sequence alignments of Li: *Leishmania infantum* LINJ_36_4190 (GenBank ID: FR796468.1), Ld: *L. donovani* LDBPK_364190 (GenBank ID: FR799623.2), Lmx: *L. mexicana* LMXM_36_3991 (GenBank ID: FR799573.1), and Lb: *L. braziliensis* LBRM_35_4231 (GenBank ID: FR799010.1). Matches are highlighted in grey and the discrepancies are white, traced lines mean the gaps. Source: BLASTN 2.6.1+ (https://blast.ncbi.nlm.nih.gov/Blast.cgi).

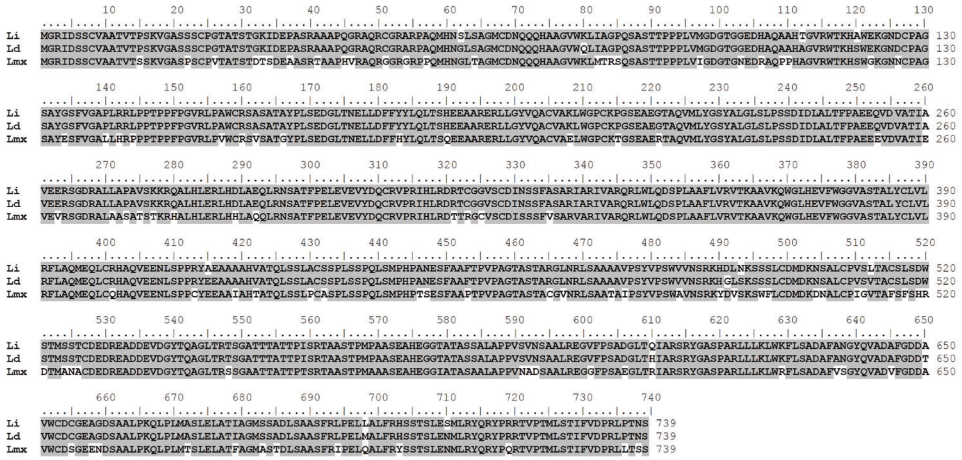

Fig. 1B: multiple peptide sequence alignments of Li: *Leishmania infantum* hypothetical protein LINJ_36_4190 (GenBank ID: XP_001469615.1), Ld: *L. donovani* hypothetical protein LDBPK_364190 (GenBank ID: XP_003865533.1) and Lmx: *L. mexicana* LMXM_36_3991 (GenBank ID: XP_003874756.1). Matches are highlighted in grey and the discrepancies are white, traced lines mean the gaps. Source: BLASTX 2.6.1+ (https://blast.ncbi.nlm.nih.gov/Blast.cgi).

EPITOPE PREDICTION RESULTS

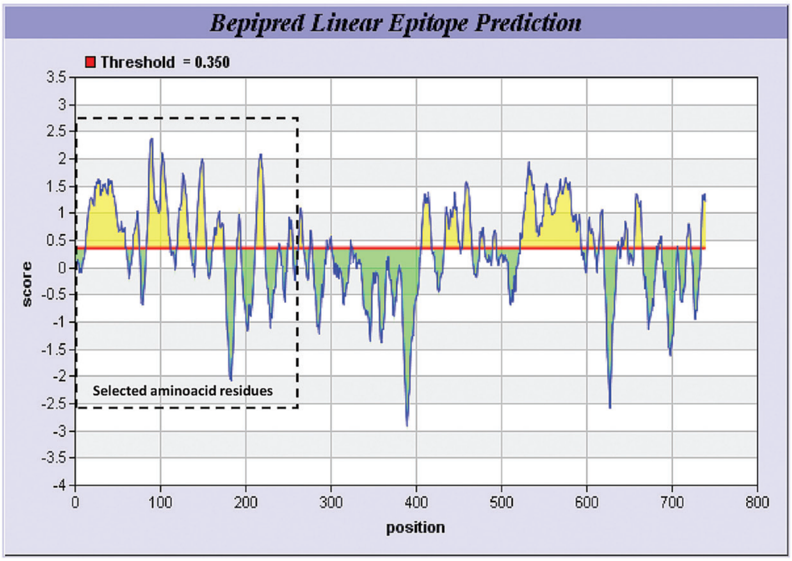

Fig. 2: epitopes prediction to B-cell using data base IEDB Analysis Resource. Predict epitope regions are plotted up the threshold of 0.350 (red line) and are highlighted in yellow. Abscissa refers to amino acids positions. Protein encoding by Lc36 gene has 733 amino acids. Dashed line corresponds to the fragment selected for the immunoassays of the Lc36 protein, corresponding to the region between 1-255 amino acid residues. Source: IEDB Analysis Resource, URL http://tools.immuneepitope.org/bcell/.

TABLE I

Predicted peptides. Epitopes prediction to B-cell using data base IEDB Analysis Resource

No.	Start	End	Peptide	Length
1	12	59	TVTPSKVGASSSCPGTATSTGKIDEPASRAAAPQGRAQRCGRARPAQM	48
2	68	75	CDNQQQHA	8
3	84	111	GPQSASTTPPPLVMGDGTGGEDHAQAAH	28
4	120	135	AWEKGNDCPAGSAYGS	16
5	142	153	RRLPPTPPFPGV	12
6	162	174	ASATAYPLSEDGL	13
7	190	194	HEEAA	5
8	212	222	PCKPGSEAEGT	11
9	239	240	SS	2
10	249	255	PAEEQVD	7
11	262	267	EERSGD	6
12	275	277	VSK	3
13	295	296	RN	2
14	298	300	ATF	3
15	323	325	CGG	3
16	406	417	EENLSPPRYAEA	12
17	433	451	SPLSSPQLSMPHPANESFA	19
18	453	465	FTPVPAGTASTAR	13
19	475	479	AVPSY	5
20	490	493	HDLN	4
21	522	593	TMSSTCDEDREADDEVDGYTQAGLTRTSGATTTAT TPISRTAASTPMPAASEAHEGGTATASSALAPPVSVN	72
22	601	608	VFPSADGL	8
23	615	619	RYGAS	5
24	636	638	FAN	3
25	641	648	QVADAFGD	8
26	656	665	GEAGDSAALP	10
27	682	682	M	1
28	684	687	SADL	4
29	706	706	T	1
30	717	721	YPRRT	5

Source: IEDB Analysis Resource, URL http://tools.immuneepitope.org/bcell/. Larsen et al. (2006).

```
Calculating prediction for the following proteins
with reference library 08:
>pLc36
... Done.
*** List of predicted non-TM-protein codes ***
>pLc36
*** List of predicted TM-protein codes ***
none
=== Result of the prediction ===
>pLc36
# TMH:  1 Q:  0.58 !!! Warning! Non-TM protein!
@  385   2.698 core:  383 ..  388 7.791e-02
<-------- end of list -------->
```

Fig. 3: prediction of transmembrane domain of the pLc36 protein. Source: author, using DAS-TMfilter. Available from: http://mendel.imp.ac.at/sat/DAS/DAS.html.

CLONING AND EXPRESSION IN *ESCHERICHIA COLI*

DNA of *Leishmania infantum* was extracted from a culture of promastigotes by the Pure Link® Kit DNA extraction (Life Technologies). Amplification of the parasite *Lc36* gene was performed using primers Lc36 Forward (5'-AAACCATGGGGCGAATCGACTCCTCTTGTG-3') and Lc36 Reverse (5'-TTTAAGCTTGTCCACCT-GCTCCTCGGCCG-3') containing *Nco*I and *Hind*III restriction enzyme site sequences, respectively. For amplification, 2.5 U of the High Fidelity *Pfu* DNA Polymerase (Thermo Scientific™). The reaction consisted of an initial denaturation step at 95ºC for 2 min followed by 30 cycles of denaturation at 95ºC for 1 min, annealing at 60ºC for 1 min, and extension at 72ºC for 1 min, followed by a final extension at 72ºC for 5 min. The polymerase chain reaction (PCR) product was purified using the purification kit GFX ™ (GE Healthcare) and the concentration of the DNA was measured using the NanoDrop Lite Spectrophotometer (Thermo Scientific). The amplified fragment was ligated into the pGEM-T easy vector (Promega). After *Escherichia coli* DH5α transformation, the selected transformants were confirmed by DNA restriction analysis. The insert of the plasmid DNA was then liberated with *Nco*I and *Hind*III and ligated using these same sites into the pET28a expression vector (Novagen). In order to check if the DNA fragment was cloned in the correct frame, the recombinant plasmid named pET28a_Lc36 was subsequently sequenced by Macrogen Inc. prior to transformation of *E. coli* BL21 (DE3) cells.

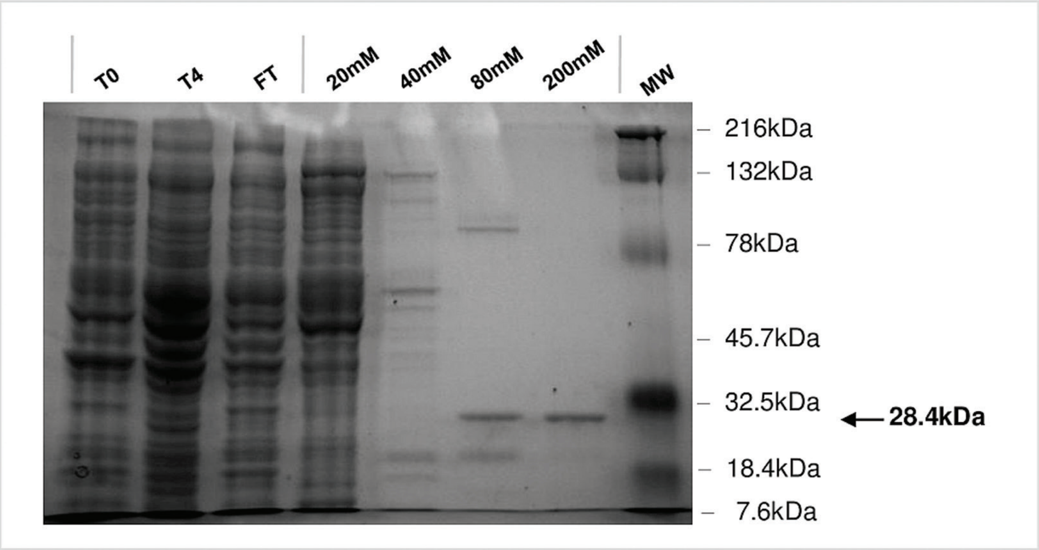

Fig. 4: the rLc36 expression in *Escherichia coli* and purification are confirmed by SDS-PAGE and western blot analysis. A 12% polyacrylamide gel was used and proteins stained with Coomassie blue. Lanes contain proteins from a cell extract of *E. coli* BL21(DE3) pET28aLc_36. T0: total lysate before induction; T4: total lysate 4 h after induction; FT: total flow through in affinity chromatography; followed by washings of the nickel column with imidazole solutions of 20 mM, 40 mM, 80 mM and 200 mM. MW: Protein molecular weight (Kaleidoscope Prestained Standards - Bio-Rad). The arrow indicates the molecular weight of the recombinant protein rLc36. Source: authors.

REAL-TIME QUANTITATIVE PCR (RT-QPCR) RESULTS

Equation 1
Plasmid DNA template size equation:

$$(1)\ n = (c \times [t \times 660]) \times (6.022 \times 10^{23})$$

Where, n = cDNA copy numbers/µL, c = plasmid concentration in g/µL, t = plasmid size (vector+insert) in base pairs, average molecular weight double-stranded DNA molecule = 660 g/mol, Avogadro constant = 6.022 x 10^{23} molecules/mol (Applied Biosystems tutorials, http://www6.appliedbiosystems.com/support/tutorials/pdf/quant_pcr.pdf).

TABLE II

Real-time quantitative polymerase chain reaction (RT-qPCR) absolute standard curve padronisation using recombinant plasmid pET28a+_Lc36 and cDNA from *Leishmania infantum* promatigotes and intercellular amastigotes

Assay	pDNA/cDNA	Amount (ng)	Average C_T	Copy number / µL	Copy number / reaction (10 µL)	Curve parameters
	1_pDNA	88,7	4,44±0,05	1066860405	10668604047	Slope: -3,34
Standard	2_pDNA	8,87	5,31±0,05	106686040	1066860405	Effciency: 99,23%
Curve	3_ pDNA	0,887	8,90±0,07	10668604	106686040,5	R^2: 0,98
1:10	4_ pDNA	0,0887	12,79±0,08	1066860,4	10668604,05	Threshold: 0,324
	5_ pDNA	0,00887	16,65±0,18	106686,04	1066860,405	Tm: 83ºC
	6_ pDNA	0,000887	20,28±0,22	10668,604	106686,0405	
	Am_50_18h	50	36,03±0,84	0,14601325	1,4601325	
	Am_50_48h	50	32,19±0,60	2,051866929	20,51866929	
Samples	Am_50_72h	50	Undetermined	-	-	
	MØunifected	50	Undetermined	-	-	
	Pro_50_FinalLog	50	Undetermined	-	-	

Am: amastigote cDNA (18 h, 48 h and 72 h post-infection); MØ: uninfected macrophage cDNA; Pro: logarithmic phase promastigote cDNA; C_T: cycle threshold; Tm: melting temperature. Plots illustrated at Figs 4 and 5. Source: author, adapted by Origin® and Excel® using file from software StepOne™ v2.3.

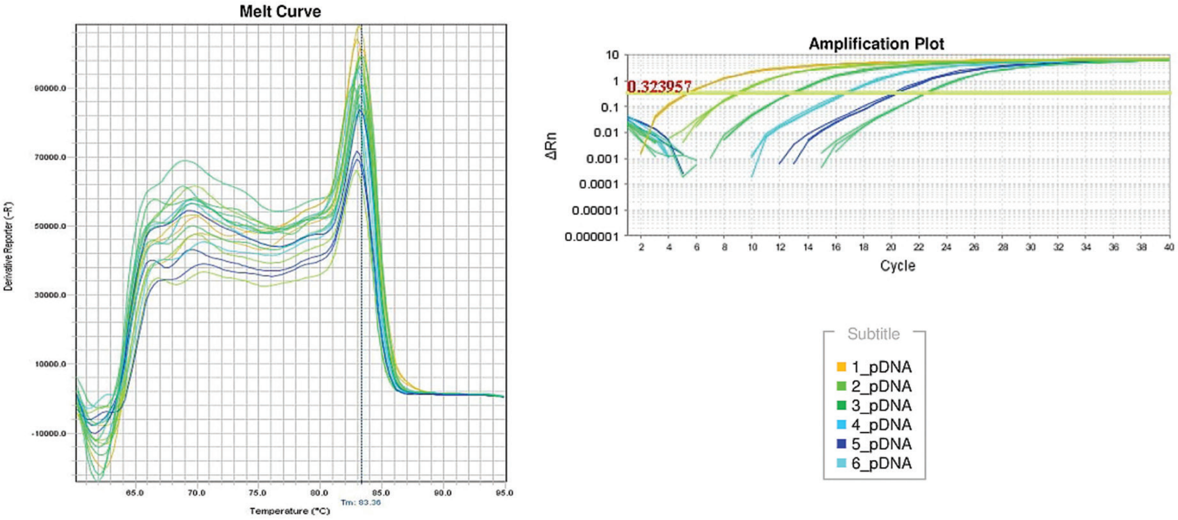

Fig. 5: amplification and melt plots of standard curve of the Lc36 expression real-time quantitative polymerase chain reaction (RT-qPCR) assay by absolute quantification. Plots data are described in Table II. Source: author, software StepOne™ v2.3.

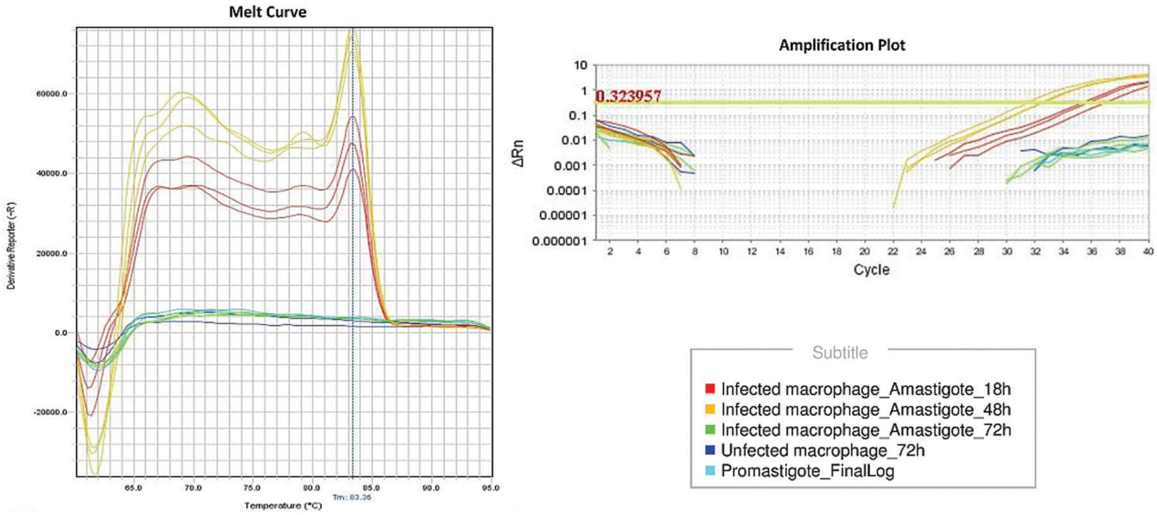

Fig. 6: amplification and melt plots of Samples of the Lc36 expression real-time quantitative polymerase chain reaction (RT-qPCR) assay by absolute quantification. Plots data are described in Table II. Source: author, software StepOne™ v2.

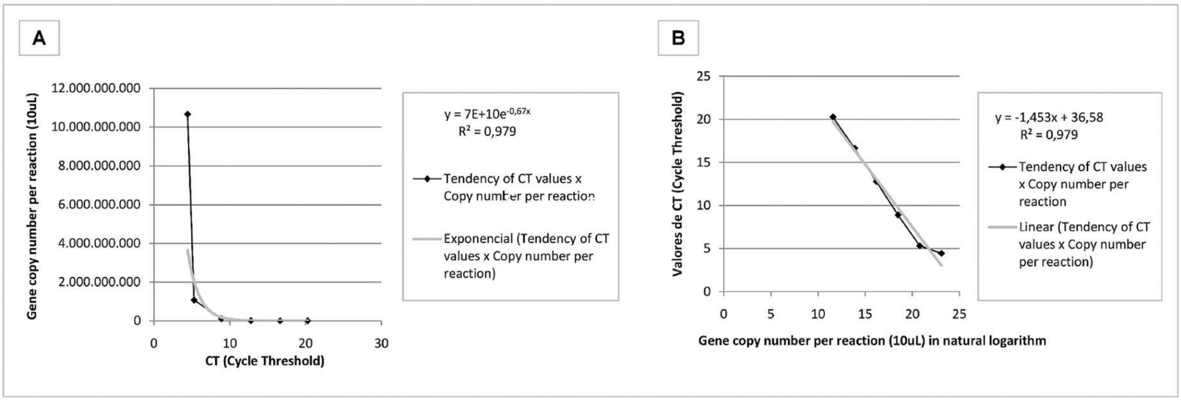

Fig. 7: set equations for Lc36 gene expression evaluation. (A) Plot of exponential tendency curve of copy number per reaction related to standard absolute curve C_T values. (B) Plot of natural logarithmic of copy number per reaction related to standard absolute curve C_T values. Source: author, adapted by Origin® and Excel® using file from software StepOne™ v2.3.

IMMUNOASSAYS RESULTS

Equations 2, 3 and 4. Cut-off and accuracy determination:

According to Frey et al. (1998), for *cut-off* calculation, we used the sum of average plus standard deviation of 20 known-negative sera absorbance (Supplementary data, Tables III-IV) that showed the lowest values during our enzyme-linked immunosorbent assay (ELISA) assays using rLc36 selected fragment protein expressed. That sum was multiplied by an appropriate constant for *cut off* determination, which were 1.772 for 95% of confidence and 2.602 for 99% of confidence. As sera were previously confirmed for canine visceral leishmaniasis (CVL), we separate them into true positives (TP) and true negatives (TN), as well as false positives (FP) and false negatives (FN) groups. Sera absolute amount of each group was used for sensibility (s) and specificity (e) calculation according to equations (2) s = TP/(TP + FN) and (3) e = TN/(TN + FP), respectively. Accuracy (Ac) was calculated using equation (4) Ac = e + p.(s-e), in which p is prevalence and was determined by the relation between amount of dog sera infected and total of amount dog sera (p = Do/n), according to Kawamura (2002).

TABLE III

Absorbance average of sera tested against recombinant protein Lc36 after 60 min of reaction. Sera dilution of 1:200

Sera identification	Absorbance (405 nm)	Sera identification	Absorbance (405 nm)	Sera identification	Absorbance (405 nm)	Sera identification	Absorbance (405 nm)
n1	0.2565	*n17*	0.1725	*n38*	**0.0365**	*n56*	**0.1335**
n2	0.1425	*n18*	0.1445	*n40*	**0.1075**	*n57*	0.1945
n4	0.2265	*n19*	0.1415	*n41*	**0.0675**	*n58*	**0.0525**
n5	0.2005	*n21*	0.1345	*n42*	0.1485	*n59*	**0.0255**
n6	0.1575	*n23*	**0.1155**	*n43*	**0.0485**	*n60*	0.1385
n7	0.2035	*n24*	**0.0625**	*n44*	0.2465	*p2*	0.413
n8	**0.0865**	*n25*	**0.1125**	*n45*	**0.0605**	*p6*	0.204
n9	**0.0885**	*n29*	0.183	*n46*	0.2095	*p7*	0.234
n10	0.2135	*n30*	0.1505	*n47*	**0.0295**	*p8*	0.534
n11	0.2365	*n31*	0.1375	*n48*	**0.0375**	*p11*	0.247
n12	0.2195	*n33*	0.2475	*n49*	**0.1205**	*p15*	0.218
n13	0.1335	*n34*	0.1355	*n51*	**0.0535**	*p16*	1.132
n14	0.2035	*n35*	0.2265	*n52*	0.1695	*p17*	0.209
n15	**0.0795**	*n36*	0.1435	*n53*	**0.1135**	*p18*	0.101
n16	0.1765	*n37*	**0.0285**	*n54*	0.2525	*p19*	0.332
p20	0.327	*p38*	0.232	*p55*	0.207	*p76*	0.098
p21	0.347	*p39*	0.559	*p56*	0.242	*p77*	0.283
p22	0.558	*p40*	0.553	*p58*	0.211	*p80*	0.201
p23	0.236	*p41*	0.12	*p59*	0.142	*p81*	0.35
p24	0.278	*p42*	0.236	*p60*	0.367	*p82*	0.355
p25	0.706	*p43*	0.663	*p61*	0.287	*p83*	0.187
p26	1.314	*p44*	0.844	*p62*	0.335	*p84*	0.301
p28	0.268	*p45*	2.025	*p63*	0.23	*p85*	1.162
p29	0.364	*p46*	0.327	*p64*	0.967	*p86*	0.628
p30	0.258	*p47*	0.195	*p65*	0.184	*p87*	0.263
p31	0.231	*p48*	0.697	*p68*	0.22	*p88*	0.55
p32	0.638	*p49*	0.885	*p69*	0.38	*p89*	0.63
p33	1.443	*p50*	0.426	*p72*	0.298	*p91*	0.377
p35	0.129	*p51*	0.556	*p73*	0.382	*p92*	0.259
p36	0.39	*p52*	0.19	*p74*	0.154	*p93*	0.301
p37	0.504	*p53*	1.314	*p75*	0.127	*p94*	0.162

Sera name started by letter *n* indicate negative sera for visceral leishmaniasis (VL) and started by p, indicate positive sera for VL. Values bold highlighted were used for *cut off* determination. Blank average: 0.1065. Source: author.

TABLE IV

Absorbance average of visceral leishmaniasis negative against recombinant protein Lc36 after 60 min of reaction. Sera dilution of 1:200

Sera identification	Absorbance (405 nm)	Sera identification	Absorbance (405 nm)	Sera identification	Absorbance (405 nm)	Sera identification	Absorbance (405 nm)
n1b	**0,0975**	*n16b*	0,1495	*n31b*	**0,0395**	*n46b*	0,1385
n2b	0,1505	*n17b*	**0,0515**	*n32b*	0,1005	*n47b*	0,2015
n3b	0,1965	*n18b*	**0,0305**	*n33b*	**0,0555**	*n48b*	**0,0475**
n4b	0,2055	*n19b*	**0,0495**	*n34b*	0,1295	*n49b*	**0,0205**
n5b	0,1575	*n20b*	0,1095	*n35b*	**0,0415**	*n50b*	0,1415
n6b	0,2035	*n21b*	**0,0585**	*n36b*	0,1975	-	-
n7b	**0,0745**	*n22b*	0,1035	*n37b*	**0,0455**	-	-
n8b	0,0535	*n23b*	0,1915	*n38b*	0,2005	-	-
n9b	0,2115	*n24b*	0,1525	*n39b*	**0,0235**	-	-
n10b	0,2085	*n25b*	0,1275	*n40b*	**0,0325**	-	-
n11b	0,1985	*n26b*	0,1445	*n41b*	0,1125	-	-
n12b	**0,0405**	*n27b*	**0,0425**	*n42b*	**0,0615**	-	-
n13b	0,1945	*n28b*	0,1255	*n43b*	0,1595	-	-
n14b	**0,0905**	*n29b*	0,1445	*n44b*	0,1045	-	-
n15b	0,1985	*n30b*	**0,0305**	*n45b*	0,1925	-	-

Letter b (n1b) indicates sera are different to assay sensibility and specificity sera (Table III). Values bold highlighted were used for *cut off* determination. Blank average: 0.1005. Source: Author.

TABLE V

Characteristics of 39 dogs with positive diagnosis for visceral leishmaniasis

			RIFI		Parasitological			PCR (*Leishmania* spp.)			PCR (*L. chagasi*)		
Sera identification	Absorbance 405 nm*	Clinical signs	Screenig	Tit	Blood	MO**	Lymphnode	Blood	MO**	Linfonodo	Blood	MO**	Linfonodo
p42b	1,399	Present	Positive	40	Positive	Positive	Negative	Positive	Positive	Positive	Negative	Positive	Positive
p43b	0,321	Present	Positive	640	Positive	Positive	Positive	Positive	Positive	Positive	Negative	Positive	Positive
p44b	0,354	Present	Positive	640	Negative	Negative	Negative	Positive	Positive	Positive	Negative	Positive	Negative
p45b	0,323	Present	Positive	320	Positive	Negative	Positive	Negative	Positive	Positive	Negative	Negative	Positive
p46b	0,459	Present	Positive	640	Negative	Negative	Negative	Positive	Positive	Positive	Positive	Positive	Positive
p47b	0,394	Present	Positive	160	Negative	Positive	Positive	Positive	Positive	Positive	Negative	Positive	Negative
p48b	0,427	Present	Positive	160	Negative	Negative	Positive	Positive	Positive	Positive	Positive	Positive	Positive
p49b	0,366	Present	Positive	640	Negative	Negative	Positive	Positive	Positive	Negative	Positive	Positive	Negative
p50b	1,045	Absent	Positive	320	Negative	Positive	Positive	Positive	Positive	Negative	Positive	Positive	Negative
p51b	0,292	Present	Positive	640	Negative	Positive	Negative	Positive	Positive	Positive	Negative	Positive	Positive
p52b	0,325	Present	Positive	640	Negative	Negative	Negative	Positive	Positive	Positive	Negative	Positive	Positive
p53b	0,491	Present	Positive	640	Negative	Negative	Positive	Positive	Positive	Positive	Negative	Positive	Positive
p54b	0,387	Present	Positive	640	Negative	Positive	Negative	Positive	Positive	Positive	Negative	Positive	Positive
p55b	0,488	Present	Positive	640	Negative	Negative	Positive	Positive	Positive	Positive	Positive	Positive	Positive
p56b	0,265	Present	Positive	640	Negative	Positive	Positive	Positive	Positive	Positive	Positive	Positive	Positive
p57b	0,39	Present	Positive	640	Negative	Negative	Negative	Positive	Positive	Positive	Positive	Positive	Positive
p58b	0,311	Present	Positive	320	Negative	Negative	Negative	Positive	Positive	Positive	Positive	Positive	Positive
p59b	0,46	Present	Positive	640	Negative	Negative	Positive	Positive	Positive	Positive	Positive	Positive	Positive
p60b	0,471	Present	Positive	320	Negative	Negative	Negative	Positive	Positive	Positive	Negative	Positive	Positive
p61b	0,292	Present	Positive	640	Negative	Negative	Positive	Positive	Positive	Positive	Positive	Positive	Positive
p62b	0,407	Present	Positive	640	Negative	Negative	Negative	Positive	Positive	Positive	Negative	Positive	Positive
p63b	1,268	Present	Positive	160	Negative	Negative	Positive	Positive	Positive	Positive	Positive	Positive	Positive
p64b	0,734	Absent	Positive	640	Negative	Negative	Negative	Positive	Positive	Negative	Negative	Positive	Positive
p65b	0,393	Present	Positive	640	Negative	Positive	Positive	Positive	Positive	Positive	Positive	Positive	Positive
p66b	0,654	Present	Positive	320	Negative	Negative	Negative	Positive	Positive	Positive	Negative	Positive	Positive
p67b	0,792	Present	Positive	160	Negative	Negative	Negative	Positive	Positive	Negative	Negative	Positive	Negative
p68b	0,478	Present	Positive	320	Negative	Negative	Negative	Positive	Positive	Positive	Negative	Positive	Positive
p69b	0,383	Absent	Positive	80	Negative	Positive	Positive	Positive	Positive	Positive	Negative	Positive	Positive
p70b	0,413	Absent	Positive	80	Positive	Positive	Negative	Positive	Positive	Negative	Positive	Negative	Negative
p71b	0,263	Absent	Positive	640	Negative	Negative	Positive	Positive	Positive	Positive	Positive	Positive	Positive
p72b	0,243	Present	Positive	80	Negative	Negative	Negative	Positive	Positive	Positive	Negative	Positive	Negative
p73b	0,199	Present	Positive	640	Negative	Negative	Negative	Positive	Positive	Negative	Negative	Positive	Positive
p74b	0,193	Present	Positive	320	Positive	Positive	Negative	Positive	Positive	Negative	Positive	Positive	Negative

			RIFI		Parasitological			PCR (*Leishmania* spp.)			PCR (*L. chagasi*)		
Sera identification	Absorbance 405 nm*	Clinical signs	Screenig	Tit	Blood	MO**	Lymphnode	Blood	MO**	Linfonodo	Blood	MO**	Linfonodo
p75b	0,174	Present	Positive	640	Negative	Negative	Negative	Positive	Positive	Positive	Negative	Positive	Positive
p76b	0,172	Present	Positive	640	Negative	Negative	Negative	Positive	Positive	Negative	Negative	Positive	Positive
p77b	0,16	Present	Positive	640	Positive	Negative	Positive	Positive	Positive	Negative	Positive	Positive	Negative
p78b	0,148	Absent	Positive	40	Negative	Negative	Negative	Positive	Positive	Negative	Positive	Positive	Negative
p79b	0,137	Present	Positive	640	Negative	Negative	Negative	Positive	Positive	Negative	Negative	Positive	Negative
p80b	0,134	Present	Positive	640	Negative	Negative	Negative	Positive	Positive	Positive	Positive	Positive	Positive

*: absorbance values with the mean of the subtracted blank; **: MO = macrophages. Sera marked in gray highlight the asymptomatic animals. Letter b (p1b) indicates sera are different to assay sensibility and specificity sera (Table III). Blank average: 0.1005. Source: FMVZ - UNESP and Author.
